# Exploring Antibiotic Resistance Diversity in *Leuconostoc* spp. by a Genome-Based Approach: Focus on the *lsa*A Gene

**DOI:** 10.3390/microorganisms9030491

**Published:** 2021-02-26

**Authors:** Elisa Salvetti, Ilenia Campedelli, Ilaria Larini, Giada Conedera, Sandra Torriani

**Affiliations:** 1 Department of Biotechnology, University of Verona, 37134 Verona, Italy; elisa.salvetti@univr.it (E.S.); ilaria.larini@univr.it (I.L.); conederagiada93@gmail.com (G.C.); 2 Microbion srl, San Giovanni Lupatoto, 37057 Verona, Italy; i.campedelli@microbion.it

**Keywords:** *Leuconostoc*, food chain, genomes, antibiotic resistance, *lsa*A gene, clindamycin, quinupristin-dalfopristin

## Abstract

*Leuconostoc* spp. are environmental microorganisms commonly associated with fermented foods. Absence of antibiotic resistance (AR) in bacteria is a critical issue for global food safety. Herein, we updated the occurrence of AR genes in the *Leuconostoc* genus through in silico analyses of the genomes of 17 type strains. A total of 131 putative AR traits associated with the main clinically relevant antibiotics were detected. We found, for the first time, the *lsa*A gene in *L. fallax* ATCC 700006^T^ and *L. pseudomesenteroides* NCDO 768^T^. Their amino acid sequences displayed high similarities (59.07% and 52.21%) with LsaA of *Enterococcus* *faecalis* V583, involved in clindamycin (CLI) and quinupristin-dalfopristin (QUD) resistance. This trait has different distribution patterns in *Leuconostoc* nontype strains—i.e., *L. pseudomesenteroides*, *L. lactis* and *L. falkenbergense* isolates from fermented vegetables, cheeses, and starters. To better explore the role of *lsa*A, MIC for CLI and QUD were assessed in ATCC 700006^T^ and NCDO 768^T^; both strains were resistant towards CLI, potentially linking *lsa*A to their resistant phenotype. Contrarily, NCDO 768^T^ was sensitive towards QUD; however, expression of *lsa*A increased in presence of this antibiotic, indicating an active involvement of this trait and thus suggesting a revision of the QUD thresholds for this species.

## 1. Introduction

*Leuconostoc* spp. belong to the functional group of Lactic Acid Bacteria (LAB) and are generally found on green vegetation and roots; from this natural habitat, they disseminate to various plant niches, such as lychee fruits (*L. litchi* [1]), palm wine (*L*. *palmae* [2]) cabbage and rutabaga (*L. rapi* [3]), cucumbers, carrots, olives, silage, soybeans (*L. mesenteroides* strains [4,5]) coffee beans (*L. holzapfelii* [6]) and quinoa samples (*L. lactis* and *L. mesenteroides* isolates, [7]) where they can actively participate in the fermentation process. In particular, *Leuconostoc* spp. are added as starters cultures for the production of sauerkraut and kimchi, from which the type strains of *L. fallax* [8], *L. kimchii* [9], *L. inhae* [10], *L. mesenteroides* subsp. *jonggajibkimchii* [11], and *L*. *miyukkimchii* [12], have been isolated. 

*Leuconostoc* spp. have been also associated with foods of animal origin including dairy (*L. citreum, L. lactis*, *L. mesenteroides*, *L. falkebergense* [13,14]), fish (i.e., *L. mesenteroides* in molluscs, [5]) and both in fresh and fermented meat products (*L. carnosum*, *L. citreum*, *L. mesenteroides* and *L. gelidum*; [15,16,17]). In particular, their impact on dairy technology is mainly related to their ability to produce CO_2_, which is responsible for the eye formation in some cheeses, and other aroma compounds, such as diacetyl and acetoin, thus significantly contributing to the flavor of the final product [18].

Due to their presence in fermented foods and associated history of safe consumption, leuconostocs are Generally Regarded As Safe (GRAS) microorganisms by the US Food and Drug Administration (FDA); further, some species have the Qualified Presumption of Safety (QPS) status by the European Food Safety Authority (EFSA) (https://zenodo.org/record/3828466#.YAhMB-lKgc8; accessed in 26 February 2021) and are also included in the Inventory of Microbial Food Cultures with Safety Demonstration in Fermented Food Products by the International Dairy Federation [13]. 

Despite their important roles and safety in the production of fermented foods, these strains have been also associated with negative aspects on human health, including the production of undesirable compounds, such as biogenic amines [19], and the expression of antimicrobial resistance mechanisms [20]. *Leuconostoc* spp. have been frequently found in infections caused by multiple microorganisms in patients following vancomycin treatment, towards which they are intrinsically resistant due to the presence of pentadepsipeptide with D-Lactate at the C-terminal in the peptidoglycan instead of a D-Alanine [18]. Generally, leuconostocs are susceptible to beta-lactams (ampicillin and penicillin [21]), and to antibiotics inhibiting protein synthesis (i.e., erythromycin, chloramphenicol, clindamycin and tetracycline [22,23]). However, resistant phenotypes were detected in strains of *L. pseudomesenteroides*, *L. citreum* and *L. mesenteroides* isolated from table olives and dairy products towards chloramphenicol, ciprofloxacin, clindamycin, erythromycin, gentamicin, kanamycin, oxacillin, nitrofurantoin, rifampicin, streptomycin, tetracycline, teicoplanin, trimethoprim and virginiamycin [20,21,22,24,25]; further, acquired resistance to tetracycline and erythromycin has been reported for *L. mesenteroides* strains from artisanal cheeses [20]. 

From this perspective, the present study aimed to explore the occurrence of putative antibiotic resistance genes (ARGs) in the genomes of 17 *Leuconostoc* type strains through in silico analyses. This genome-wide comprehensive survey allowed the detection of the *lsa*A gene, responsible for clindamycin and quinupristin-dalfopristin resistance, whose distribution pattern was further investigated in *L. pseudomesenteroides, L. fallax* and related strains, and its role in antibiotic resistance was phenotypically validated. The critical evaluation of genotypic and phenotypic data as it is performed within this study may represent an essential step to counteract the horizontal spread of antibiotic resistance (AR) traits from food-grade technological *Leuconostoc* strains towards foodborne commensals and other pathogenic bacteria. 

## 2. Materials and Methods

### 2.1. Genome Sequence Analysis to Retrieve the Antibiotic Resistance Genes

The genome sequences of the 17 available type strains and their annotations for the *Leuconostoc* genus (13 species and 4 subspecies) were downloaded from NCBI using the accession number reported in Table 1. The annotated sequences were employed to query the Comprehensive Antibiotic Resistance Database (CARD, version 3.1.0, https://card.mcmaster.ca/download, protein homolog model; downloaded in December 2020) [26], through the Basic Local Alignment Search Tool (BLAST, https://blast.ncbi.nlm.nih.gov, accessed in December 2020) in order to identify all AR genes. A trait was annotated as a putative AR determinant according to its BLASTp hit in CARD with a threshold of amino acid sequence identity > 30%, e-value < 1 × 10^−5^ and query coverage > 70% [27]. In addition, the amino acid sequences of all ARGs retrieved from CARD, resulting in a reference dataset of 2702 amino acid sequences (including aminoglycosides, lincosamides, macrolides, streptogramins, tetracyclines, phenicols, β-lactams, glycopeptides and folate pathway inhibitors, such as trimethoprim) were aligned against the annotated genome sequences of the dataset, and the BLASTp hits were filtered as described above. To minimize putative false negative or false positive outputs, only the putative AR determinants obtained from both approaches were considered for subsequent analyses. Moreover, the obtained data were filtered for drug class, AMR genes and resistance mechanisms, selecting only recognized EFSA antibiotics and discarding efflux pumps genes, which are too generic.

### 2.2. Analysis of the lsa*A* Gene

All the amino acid sequences of *lsa*A, *lsa*B and *lsa*C retrieved in the annotated genomes were aligned with Clustal Omega online tool, fully gap-removed with Jalview (v 2.11.1.3), and then evolutionary trees were constructed with MEGAX (v 10.1.8) using the UPGMA method with default parameters [28]. 

WP_031941011.1 of *L. pseudomesenteroides* NCDO 768^T^ and WP_010008491.1 of *L. fallax* ATCC 700006^T^ were used as queries to perform a BLASTp search against the nonredundant protein sequences (nr) database in NCBI, selecting “Leuconostoc (taxid:1243)” in the “organisms” section, query coverage > 60% and identity > 50%. To further explore intraspecies similarity, WP_031941011.1 and WP_010008491.1 were used as queries in a BLASTp search with the same parameters but restricting to organism “*Leuconostoc pseudomesenteroides* (taxid:33968)” for the first one and “*Leuconostoc fallax* (taxid:1251)” for the last. The search results at the time of the study (29 January 2021) were downloaded for phylogenetic analysis, adding the LsaA protein of *Enterococcus faecalis* V583 [29]. Sequence alignments and phylogenetic tree construction were performed as described above [28]. Finally, the genomes associated with the previous BLASTp hits and the genome of *E. faecalis* V583 were downloaded from NCBI and annotated through Rapid Annotations using Subsystems Technology (RAST, http://RAST.nmpdr.org, accessed on 26 February 2021) server [30] in order to characterize 30 kpb upstream and downstream sequences flanking the *lsa*A gene. A tBLASTx alignment against genomes to search *lsa*A was performed, then a BLASTx analysis of the mobile elements detected was carried out to identify transposases or other elements that could be responsible for the transfer of this trait (File S1). 

### 2.3. Bacterial Strains and Growth Conditions

A collection of 11 type strains of the *Leuconostoc* genus was set up based on the available strains in the collection of the Dept. of Biotechnology, University of Verona (obtained from the BCCM/LMG Bacteria Collection, Ghent, Belgium and from the Spanish Type Culture Collection CECT, Valencia, Spain). *Leuconostoc* strains were grown in de Man–Rogosa–Sharpe (MRS, Fluka, Italy) medium at 27 °C for 48 h and kept in liquid cultures with 20% (w/vol) glycerol at −80 °C for long term storage.

### 2.4. Antimicrobial Susceptibility Testing

The minimum inhibitory concentrations (MICs) of clindamycin-CLI and quinupristin-dalfopristin-QUD were determined using microdilution broth methods according to Clinical and Laboratory Standard Institute (CLSI; www.clsi.org, accessed on 26 February 2021), the European Committee on Antimicrobial Susceptibility Testing (EUCAST), and ISO standard. In particular, 96-well microtiter plates containing serial two-fold dilutions of CLI and QUD antibiotics were prepared following the instructions reported by [31]. Briefly, 320 μg/mL for CLI and 160 μg/mL for QUD antibiotic stocks at different concentrations were prepared; starting from each initial stock, 10 dilutions were produced to obtain all the antibiotic solutions necessary for the preparation of the microtiter plates. Finally, 50 μL of each antibiotic solution in the 10 different concentrations were distributed in the microtiter plates. MICs were evaluated in LAB susceptibility test medium (LSM) [32], a mixed formulation containing Iso-Sensitest broth (90%) and MRS broth (10%) as described in ISO 10932 IDF 223 document and recommended by EFSA [33]. Briefly, individual *Leuconostoc* colonies were grown overnight at 27 °C in MRS broth; thus, the suspension’s turbidity was adjusted to an OD_600_ equal to 0.2, corresponding to a concentration of about 1 × 10^8^ cfu/mL. This suspension was diluted 1:100 in LSM broth, and then 50 μL of this inoculum was added to each well of the microtiter plates prepared as described above (final concentration 5 × 10^5^ cfu/mL). This test was performed in triplicate for each strain of the collection. Plates were incubated under aerobic conditions at 27 °C for 48 h. MICs were read as the lowest concentration of an antimicrobial agent at which visible growth was inhibited. Epidemiological cut-off (ECOFF) values for CLI were retrieved from [33], while breakpoint for QUD was adopted from [23] and [34]. 

### 2.5. RNA Isolation and Real-Time PCR in L. pseudomesenteroides LMG 11482^T^ (=NCDO 768^T^)

The relative quantification of the *lsa*A gene expression was performed for the strain *L. pseudomesenteroides* LMG 11482^T^ (=NCDO 768^T^). Cell cultures of this strain were grown at 27 °C in MRS broth under three different conditions: in free-antibiotic medium, in the presence of CLI (4 μg/mL) and QUD (1 μg/mL). The cells were collected in two different growth stages corresponding to OD_600_ values equal to 0.2 and 0.8. For total RNA extraction, cells were washed with 1 mL of 10 mM Tris (pH = 8), which was prepared in sterile diethyl pyrocarbonate (DEPC)-treated water. The pellet was treated with 500 μL of lysozyme (10 mg/mL) and was incubated at 37 °C for 1 h. After centrifugation (4 °C, 8000 rpm, 4 min) and elimination of the supernatant, the pellet was treated with 1 mL of Trizol solution and was incubated for 5 min at room temperature. Subsequently, 200 μL of chloroform were added and vigorously mixed. After centrifugation (4 °C, 10,000 rpm, 15 min), the supernatant was treated with 500 μL of isopropyl alcohol and left for 10 min at room temperature. Total RNA was pelleted by centrifugation at 13,000 rpm for 10 min at 4 °C, washed with 1 mL of ethanol 75%, and dissolved in 35 μL of sterile water (RNAse- and DNAse-free). The purification and transcription of the RNA was performed using, respectively, Turbo DNA-free (ThermoFisher Scientific Inc., Waltham, MA, USA) and ImProm-IITM Reverse Transcriptase (Promega, Madison, WI, USA) kit, following the manufacturer’s instructions.

All real-time PCR reactions were performed using a Light Cycler Nano (Roche, Switzerland) with a FastStart Essential DNA Green Master added with the primers 16S-F /16S-R (amplicon size: 157 pb, [35]) and lsaA-F (5′-CCCCAGACAATTCAAGACTC-3′) and lsaA-R (5′-CTCGAAAATTTGCGCCAGAG-3′), specifically designed in the present study using the Oligo Analyzer software (https://eu.idtdna.com, accessed on 26 February 2021) (amplicon size: 137 bp). The amplification program included an initial incubation at 94 °C for 6 min followed by 45 cycles at 95 °C for 30 s, 60 °C for 30 s and 72 °C for 45 s, and finally 95 °C for 10 s. At the end of the PCR, a dissociation curve was generated to verify the presence of unspecific products or primer dimers. Two independent biological replicates were performed for each growth condition and data were obtained from three technical replicates per sample. The analysis of gene expression was performed using the 2^−∆∆ct^ method [36] with 16S rRNA as internal control. 

## 3. Results

### 3.1. In Silico Prediction of ARGs in the Leuconostoc Genus

**Figure 1:**
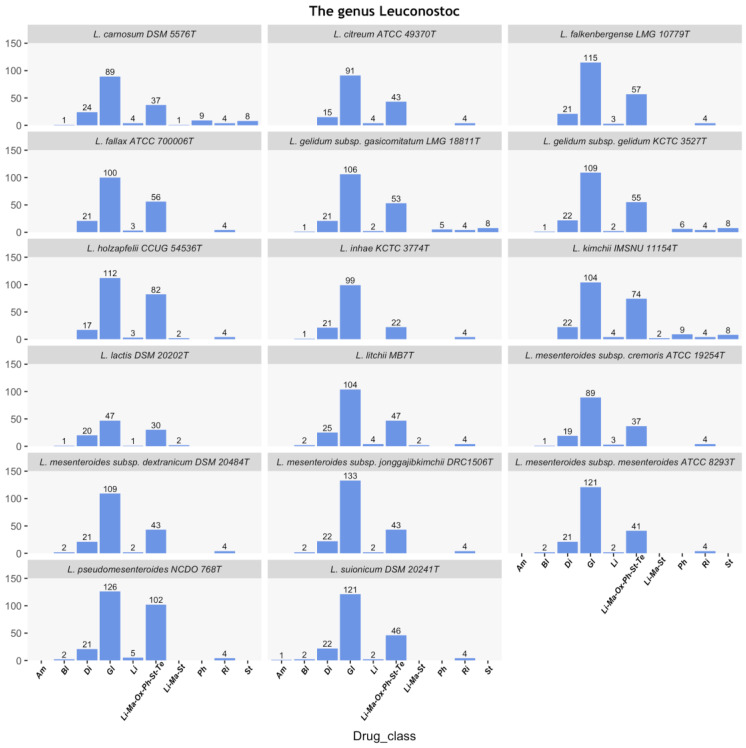
Putative antibiotic resistance (AR) genes identified in *Leuconostoc* spp. Putative antibiotic resistance genes (ARGs) detected are reported for each strain of the dataset. They are involved in the resistance towards aminoglycosides (Am), beta-lactams (Bl), diaminopyrimidines (Di), glycopeptides (Gl), lincosamides (Li), ABC-F ATP-binding cassette ribosomal protection protein ABC cassettes (Li-Ma-Ox-Ph-St-Te), erythromycin (Li-Ma-St), chloramphenicol (Ph), rifampicin (Ri) and streptogramins (St). The number of hits per drug class is reported on each barplot.

The annotated sequences of the 17 type strains of the *Leuconostoc* genus were downloaded from NCBI database and aligned against the protein sequences in CARD to retrieve all putative AR genes carried by these strains. Based on the selection criteria described in the Materials and Methods section, a total of 3197 hits belonging to 131 protein sequences were identified and they were mainly putatively involved in the resistance for aminoglycosides (Am) (n = 1), beta-lactams (Bl) (n = 1), diaminopyrimidines (Di) (n = 33), glycopeptides (Gl) (n = 46), lincosamides (Li) (n = 1), erythromycin (Li-Ma-St) (n = 6), chloramphenicol (Ph) (n = 8), rifampicin (Ri) (n = 2) and streptogramins (St) (n = 8). Moreover, several genes annotated as ABC-F ATP-binding cassette ribosomal protection protein ABC cassettes (n = 25) were retrieved due to their roles in resistance to lincosamides, macrolides, oxazolidinones, chloramphenicol, streptogramins and tetracyclines (Figure 1 and Table S1). 

The analysis revealed the presence of 1361 hits corresponding to 59 drug efflux pumps proteins in the 17 type strains examined, belonging to the Major Facilitator Superfamily (MFS) antibiotic efflux pump, Outer Membrane Porin (Opr), Resistance-Nodulation-cell Division (RND) antibiotic efflux pump, Small Multidrug Resistance (SMR) antibiotic efflux pump, ATP-Binding Cassette (ABC) antibiotic efflux pump and Multidrug And Toxic compound Extrusion (MATE) transporter (Figure S1).

Among the hits obtained, WP_031941011.1 of *L. pseudomesenteroides* NCDO 768^T^ and WP_010008491.1 of *L. fallax* ATCC 700006^T^ were found to have high similarities (51.8% and 58.9%, respectively) with the LsaA protein of *E. faecalis* ATCC 29212 in the CARD database and as such were further investigated. 

### 3.2. Distribution Patterns of the lsa*A* Gene in the Leuconostoc Genus

**Figure 2:**
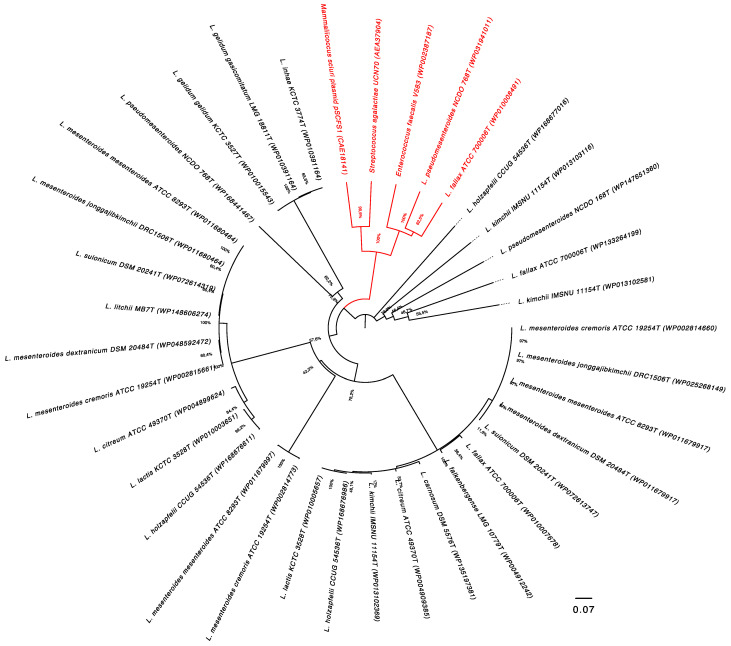
Evolutionary relationships of LsaA, LsaB and LsaC proteins in *Leuconostoc* type strains. The evolutionary history was inferred using the UPGMA method [38]. The percentage of replicate trees in which the associated taxa clustered together in the bootstrap test (1000 replicates) are shown next to the branches [39]. The tree is drawn to scale, with branch lengths in the same units as those of the evolutionary distances used to infer the phylogenetic tree. The evolutionary distances were computed using the Poisson correction method [40] and are in the units of the number of amino acid substitutions per site. All positions containing gaps and missing data were eliminated (complete deletion option). Evolutionary analyses were conducted in MEGA X [41,42] by using *Enterococcus faecalis* V583, *Mammaliicoccus sciuri* (plasmid) pSCFS1 and *Streptococcus agalactiae* UCN70 as outgroups. The phylogenetic group including LsaA of *L. pseudomesenteroides* NCDO 768^T^ and *L. fallax* ATCC 700006^T^ is highlighted in red.

ARG search results showed that all the genomes in the dataset displayed genes annotated as *lsa*A, *lsa*B and *lsa*C, coding for ATP-binding cassette. In Gram-positive bacteria, these genes code for a subgroup of ABC (ATP-binding cassette) transporters which can mediate resistance to antibiotics that bind to the 50S subunit of the ribosome, including ketolides, lincosamides, macrolides, oxazolidinones, phenicols, pleuromutilins and group A and B streptogramins [37]. To better understand the relationship between these traits, a more targeted evolutionary analysis based on the amino acid sequences of genes annotated as *lsa*A, *lsa*B and *lsa*C in the *Leuconostoc* type strains was conducted, using as references amino acid sequences of antibiotic-resistant genes *lsa*A, *lsa*B and *lsaC* of *E. faecalis* V583, *Mammaliicoccus sciuri* (plasmid) pSCFS1 and *Streptococcus agalactiae* UCN70, respectively [29]. The phylogenetic analysis revealed that the LsaA proteins in *L. pseudomesenteroides* NCDO 768^T^ and in *L. fallax* ATCC 700006^T^ are related to LsaA of *E. faecalis*, involved in CLI and QUD resistance, displaying 52% and 59% similarities (query coverage of 97% and 98%), respectively (Figure 2). 

**Figure 3:**
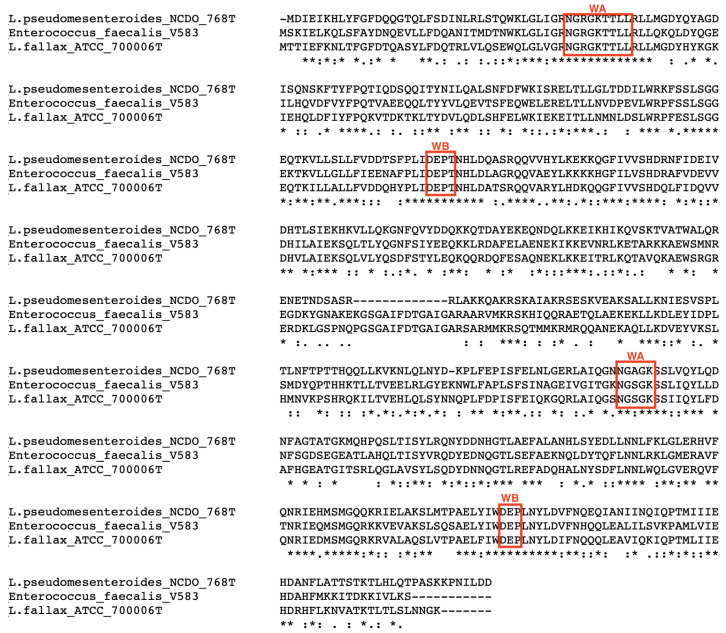
Alignment of LsaA amino acid sequence of *L. pseudomesenteroides* NCDO 768^T^, *L. fallax* ATCC 700006^T^ and *E. faecalis* V583. The red boxes highlight the Walker A (WA) and Walker B (WB) domains conserved in the three sequences.

The alignment of the LsaA amino acid sequences of the two *Leuconostoc* type strains and *E. faecalis* V583 revealed the presence of conserved Walker A and B motifs, which are peculiar for ABC transporters, and they are involved in the binding and hydrolysis of ATP [29] (Figure 3). 

**Figure 4:**
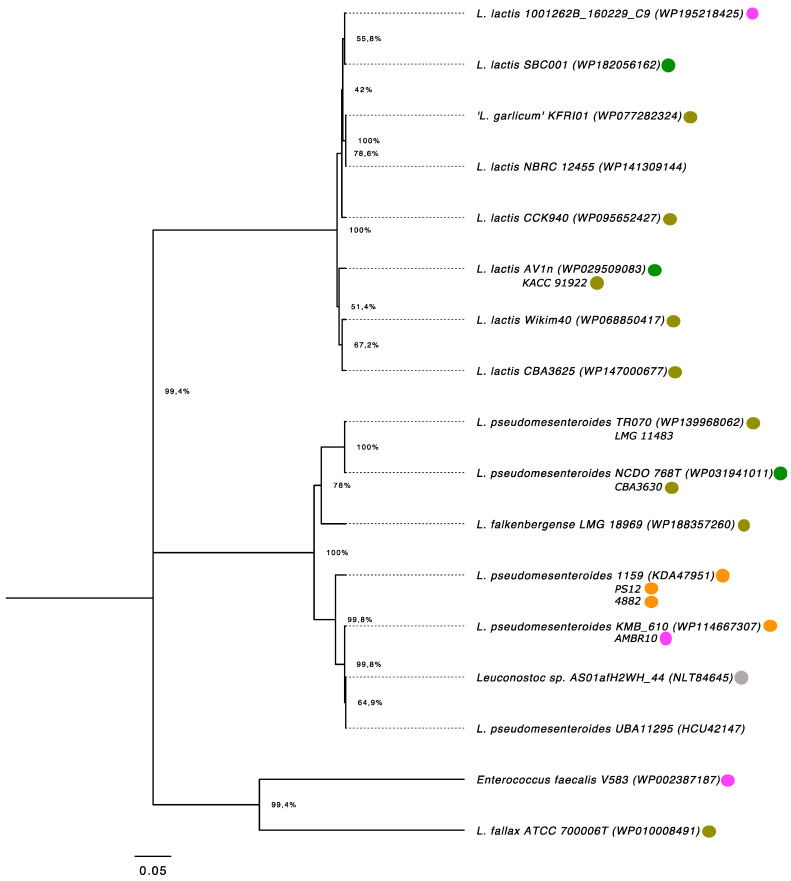
Distribution analysis of LsaA in *Leuconostoc* nontype strains. The evolutionary history was inferred using the UPGMA method [38]. The percentage of replicate trees in which the associated taxa clustered together in the bootstrap test (1000 replicates) are shown next to the branches [39]. The tree is drawn to scale, with branch lengths in the same units as those of the evolutionary distances used to infer the phylogenetic tree. The evolutionary distances were computed using the Poisson correction method [40] and are in the units of the number of amino acid substitutions per site. All positions containing gaps and missing data were eliminated (complete deletion option). Evolutionary analyses were conducted in MEGA X [41,42]. Colors refer to isolation sources: Green: raw plant material; Grey: environmental samples, Olive green: fermented foods; Orange: dairy products; Pink: clinical isolates.

In addition to its presence in the type strains of *L. pseudomesenteroides* and *L. fallax*, the analysis of distribution patterns of LsaA in the *Leuconostoc* genus (BLASTp, coverage > 60% and identity > 50%) showed that this trait occurs in 10 other strains of *L. pseudomesenteroides*. Nontype isolates are mainly associated with plant-derived fermented foods (strains TR070 and CBA3630, isolated from sourdough and kimchi, respectively), dairy products (1159 and PS12, isolated from a Danish dairy starter culture; 4882, derived from a French dairy starter culture; KMB_610, isolated from Bryndza cheese, a Slovak sheep milk cheese), clinical (AMBR10 from human adenoid) and environmental samples (AS01afH2WH_44, from the metagenomic analysis of anaerobic digestion of organic wastes). Interestingly, BLASTp analysis revealed that LsaA proteins with high similarity values were also present in seven strains of *L. lactis* mainly associated with kimchi (strains CCK940, Wikim40 and CBA3625), raw plant materials (SBC001 and AV1n, isolated from chive and fruit, respectively), and from human sources (strain 10012628_160229_C9 from fecal samples). Plant-derived fermented foods are suppliers of other strains harboring LsaA, such as *L. falkebergense* LMG 18969, associated with fermented string beans, and “*L. garlicum*” KFRI01, isolated from kimchi (Figure 4). 

### 3.3. Analysis of Flanking Regions of the lsa*A* Gene

**Figure 5:**
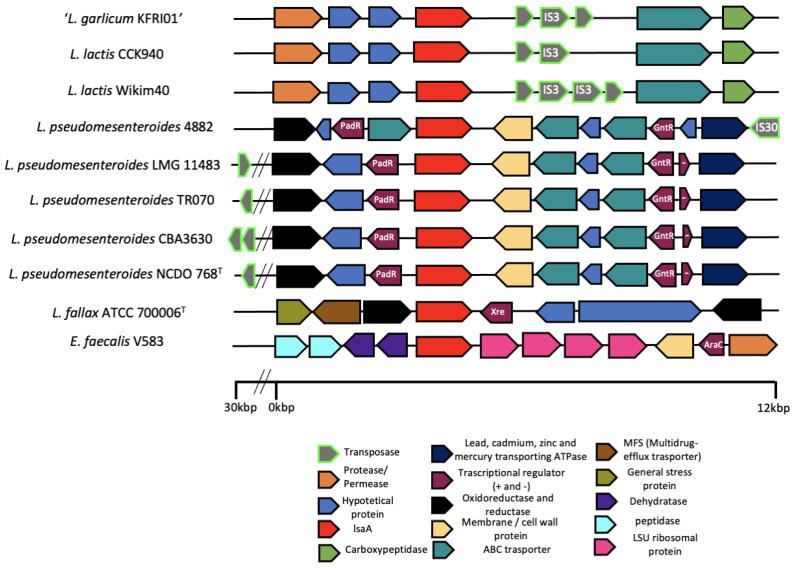
Genetic organization of the region surrounding the *lsa*A gene in nine *Leuconostoc* strains. The picture shows the genetic organization of the regions (30 kpb downstream and 30 kpb upstream) surrounding the *lsa*A gene identified in the genomes of “*L. garlicum* KFRI01”, *L. lactis* CCK940, *L. lactis* Wikim40 (in reverse strand), *L. pseudomesenteroides* 4882, *L. pseudomesenteroides* LMG 11483, *L. pseudomesenteroides* TR070, *L. pseudomesenteroides* CBA3630, *L. pseudomesenteroides* NCDO 768^T^ *L. fallax* ATCC 700006^T^ and *E. faecalis* V583.

The genome sequences of the 23 *Leuconostoc* strains harboring *lsa*A belonging to species *L. lactis*, *L. falkebergense*, *L. pseudomesenteroides*, and *L. fallax* (reported in Figure 4) were further explored to characterize the up- and downstream flanking regions of *lsa*A. Around 60 kb (30kb up- and 30 kb downstream of *lsa*A) were selected and the open reading frames (orfs) were further investigated through tBLASTn and BLASTx analyses. Strains whose genome regions exhibit putative mobile elements near to *lsa*A are depicted in Figure 5. Interestingly, the genome analysis of *L. lactis* CCK940 and Wikim40 and “*L. garlicum*” KFRI01 revealed the presence of several transposases (two copies in CCK940, three in KFRI01 and four in Wikim40) downstream of *lsa*A (between ~300 and ~1200 bp), while two genes upstream of *lsa*A were annotated as hypothetical proteins and a gene annotated as protease were found. In each strain, at least one of the genes annotated as transposases belongs to the IS3 family. Further downstream, the three genomes contained two genes reported as ABC transporter and carboxypeptidase, respectively. 

As for the other strains, mobile elements were detected in the flaking regions (but more distant compared to *L. lactis* and *L. garlicum* strains) of *lsa*A in five strains of *L. pseudomesenteroides.* More specifically, *L. pseudomesenteroides* 4882 displayed a gene annotated as a IS30 family transposase at 12 kb downstream of *lsa*A; other downstream genes between *lsa*A and IS30 were annotated as putative membrane spanning proteins, ABC transporters, hypothetical proteins, GntR transcriptional regulators and ATPase. Strains LMG 11483, TR070, CBA3630 and NCDO 768^T^ showed similar gene dispositions: a mobile element annotated as transposase (two in CBA3630) was detected at 30 kb upstream of *lsaA*, while other upstream genes were annotated as oxidoreductase, hypothetical protein and PadR transcriptional regulator; downstream of *lsa*A, genes were annotated as putative membrane proteins, ABC transporters, hypothetical proteins, GntR-like transcriptional regulators and ATPase. Sequence surrounding *lsa*A in *L. fallax* ATCC 700006^T^ included genes annotated as general stress protein, MFS multidrug efflux transporter, reductase (upstream of *lsa*A), Xre transcriptional regulator, hypothetical proteins and, again, reductase (downstream of *lsa*A). *E. faecalis* V583 displayed two genes annotated as peptidase and two genes annotated as dehydratase upstream of *lsa*A, while four genes annotated as an LSU ribosomal protein, cell wall protein, AraC transcriptional regulator and permease were found downstream of *lsa*A. 

### 3.4. Determination of Phenotypic Resistance towards CLI and QUD 

To validate the genomic data, the MIC values for CLI and QUD were determined through broth microdilution plates for 11 type strains of the *Leuconostoc* genus available in the strain collection of the Dept. of Biotechnology (University of Verona). The MIC values obtained for these two antibiotics and the relative ECOFFs are reported in Table 2. *L. fallax* LMG 13177^T^ (=ATCC 700006^T^) and *L. pseudomesenteroides* LMG 11482^T^ (=NCDO 768^T^) showed resistance to clindamycin, as they were characterized by MIC values equal to 16 and 4 μg/mL (ECOFF = 1), respectively. In addition, LMG 11482^T^ (= NCDO 768^T^) also displayed resistance towards quinupristin-dalfopristin (MIC = 8 μg/mL), while LMG 11482^T^ had an MIC = 2, which is below the cut-off value for this antibiotic (ECOFF = 4 μg/mL). 

### 3.5. Quantification of lsa*A* Expression in L. pseudomesenteroides LMG 11482^T^ (=NCDO 768^T^)

**Figure 6:**
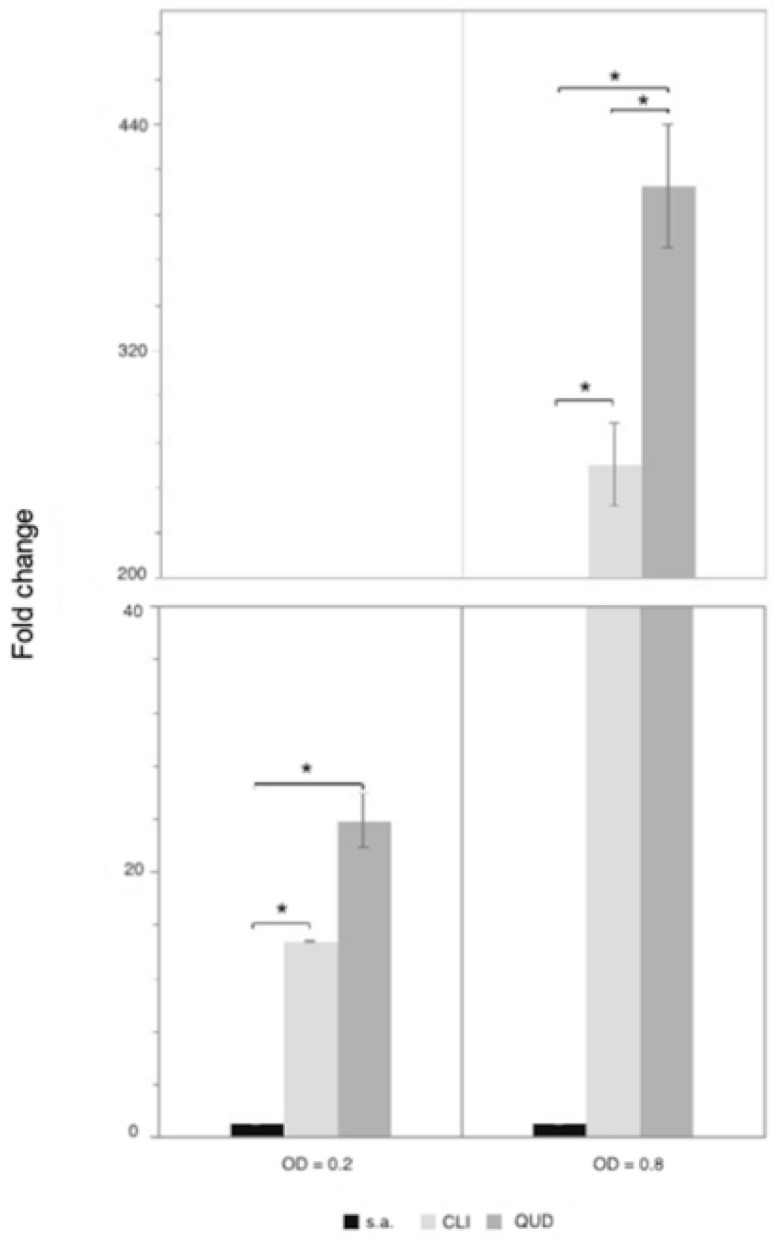
Relative quantification of *lsa*A expression for *L. pseudomesenteroides* LMG 11482^T^ (=NCDO 768^T^) grown in free-antibiotic culture (sa) in the presence of clindamycin 4 μg/mL (CLI) or quinupristin-dalphopristin 1 μg/mL (QUD). The cells were collected in the exponential and stationary growth phases 0.2 and 0.8 OD_600_, respectively. The expression of *lsa*A was normalized based on the expression of the 16S rRNA and calculated using the 2^−ΔΔct^ method. *: *p*-value < 0.05.

Since in *E. faecalis* the *lsa*A gene is associated with resistance to CLI and QUD [29], it was necessary to clarify its role in *L. pseudomesenteroides* LMG 11482^T^ (= NCDO 768^T^), which was resistant to CLI but susceptible to QUD. For this reason, the relative quantification of *lsa*A expression was performed in the presence and absence of these antimicrobial substances, using the 16S rRNA gene as internal control (Figure 6). This analysis revealed an increased expression of *lsa*A in the presence of both antibiotics either in the exponential (OD_600_ = 0.2) and stationary (OD_600_ = 0.8) growth phase of *L. pseudomesenteroides* LMG 11482^T^ (= NCDO 768^T^), indicating an involvement of *lsa*A in LMG 11482^T^ (= NCDO 768^T^) when exposed to QUD. 

## 4. Discussion

Antibiotics represent one of the largest therapeutic categories used in human and veterinary medicine for the treatment of infectious diseases caused by bacterial agents. However, overprescribing and misuse of antibiotics in medicine, aquaculture and agriculture has tremendously raised the emergence and spread of antibiotic-resistant bacteria, which constitute a critical worldwide issue in the one-health perspective where human health is directly linked to the health of animals and ecosystems [43]. In this framework, foods may act as vehicles for the transmission of AR bacteria and resistance genes along the food chain to the human gastrointestinal tract where the effectiveness of therapies could be compromised [44]. In particular, AR microorganisms can be present in food as native microbiota of raw materials, or as cultures intentionally added during food processing, or as environmental contaminants [45].

Among food-grade bacteria, *Leuconostoc* spp. have been extensively used as starter cultures and probiotics due to their long history of safe use and several species have QPS status by EFSA (https://zenodo.org/record/3828466#.YAhMB-lKgc8, accessed in 26 February 2021). However, limited information on the antimicrobial susceptibility profiles of *Leuconostoc* spp. is available, as well as their possible involvement in the dissemination of AR determinants between bacteria.

To tackle this issue, genome sequence analyses of the 17 *Leuconostoc* type strains available to date (January 2021) were carried out to improve current knowledge of the AR features of this genus. This analysis revealed the presence of 131 gene sequences putatively associated with resistance to the main important antibiotics used in medicine, including clindamycin, erythromycin, penicillins, streptogramins, and vancomycin (to which *Leuconostoc* spp. are intrinsically resistant [18]). In particular, the genome sequence analysis revealed the presence of the gene *dfr* and genes coding for penicillin-binding proteins (PBP) in all the type strains analyzed, which are involved in trimethoprim and β-lactams resistance, respectively. However, the presence of these determinants is not necessary linked to the resistance, as only mutated DHFR enzymes and PBPs are able to avoid the antibiotic effect on microbial growth [46,47]. Concerning the group of macrolides, lincosamides and streptogramins, traits detected included *erm*D, coding for an rRNA methylase [48], *vat* genes, coding for acetyltransferase enzymes [48], and *vga* and *lmr* genes coding for Major Facilitators Superfamily (MSF) transporters that pump these antibiotics out of cells [49].

Among the hits retrieved, the manual annotation and targeted similarity analysis revealed, for the first time, the presence of the *lsa*A gene in *L. pseudomesenteroides* NCDO 768^T^ and *L. fallax* ATCC 700006^T^, whose amino acid sequences displayed high similarity values (51.9% and 58.9%) with the corresponding sequence of *lsa*A in *E. faecalis* ATCC 29212 available in CARD. This gene encodes an ATP-binding cassette F (ABC-F) protein, where the ABC portions are not fused or genetically associated with transmembrane domains [37]. This genetic makeup is more closely linked to biological processes, such as DNA repair, enzyme regulation and translational control, rather than transport as conventional ABC transporters [37]. In Gram-positive pathogens (staphylococci, streptococci and enterococci), and antibiotic-producing bacteria (e.g., streptomycetes), a subgroup of ABC-F proteins mediates resistance to antibiotics that exert their action on the ribosome, including ketolides, lincosamides, macrolides, oxazolidinones, phenicols, pleuromutilins, and streptogramins of groups A and B [37]. More specifically, the *lsa*A gene was found to be responsible for the intrinsic resistance towards CLI and QUD in *E. faecalis*, where the proposed mechanism of protection was related to the ATP-energized efflux of these antibiotics out of the cell [29]. Comparison of LsaA amino acid sequence of *L. pseudomesenteroides* NCDO 768^T^ and *L. fallax* ATCC 700006^T^ with that of *E. faecalis* highlighted the presence of conserved Walker A and B ATP-binding motifs, suggesting that this gene could mediate a phenotypic resistance towards CLI and QUD in these two strains through the efflux of these antibiotics, as observed in *E. faecalis*.

Distribution analysis of this trait in the genus *Leuconostoc* revealed that other *L*. *pseudomesenteroides* nontype strains (n = 5), as well as isolates of *L. lactis* (7), *L. falkebergenes* (1) and “*L*. *garlicum*” (1) harbored *lsaA*; of these, 69.6% originated from raw or fermented foods (which are also the isolation sources of *L. fallax* [8]), corroborating the prospect that foods constitute vehicles for (putative) ARGs detected in food-grade bacteria. This scenario is further confirmed by a recent metabarcoding analysis carried out on 58 fermented foods which highlighted a large variability in both the counts per million of ARGs and in AR class frequency across the different foods and substrates and in line with the presence/absence of a starter [50]. It is interesting to note that among the strains harboring *lsa*A isolated from plant-derived fermented foods (eight strains), six of them originated from kimchi. Although Leech and colleagues [50] reported that kimchi showed few detectable ARGs compared to other substrates, a number of AR bacteria have been isolated from this food matrix, such as *Pantoea agglomerans* KM1, whose genome contains 13 antibiotic resistance genes conferring resistance to clinically important antibiotics, including penicillin G, bacitracin, rifampicin, vancomycin and fosfomycin [51]. 

As for *L. lactis*, strains SBC001, isolated from chive, and CCK940, from kimchi, have been studied for their ability to produce gluco-oligosaccharides with prebiotic and anti-inflammatory effects, while AV1n, isolated from fruit, produces high molecular weight dextrans which can have an important impact on the rheological properties of fermented products as well as immunomodulatory and antiviral activity [52]. However, no phenotypic data are available to date related to their antibiotic resistance patterns [53,54]. Contrarily, strain KACC 91922, isolated from kimchi, was characterized for its probiotic properties and thus its safety was also genotypically assessed, confirming the absence of transferable ARGs [55].

The exopolysaccharide producer strain KFRI01, isolated from kimchi [56] was allotted within the species “*L. garlicum*”, but taxonomically this species has never been validly published [57]. Therefore, a new species description, if substantiated with robust taxonomic data, should be proposed to further use this species name.

Among *L. pseudomesenteroides*, genome sequences of the strains 1159, PS12 (isolated from traditional Danish cheese starters [58]) and 4882 (from French dairy starter [59]) revealed their adaptations to dairy environments, but similarly to *L. lactis*, no phenotypic data regarding their antibiotic resistance patterns have been released. 

To evaluate the potential transferability of *lsa*A from these strains (including NCDO 768^T^ and ATCC 700006^T^) to other bacteria, the flanking regions were investigated to check for the presence of putative mobile elements. A total of 60 kb was selected (30 kb up- and downstream of *lsa*A), as it was previously observed that the transposable elements can reach a size of 52 kb [60]. *L. lactis* CCK940, Wikim40 and “*L. garlicum*” KFRI01, all isolated from kimchi, displayed from two to four copies of transposable elements right downstream of *lsa*A, including transposases of the IS3 family. Members of this family have been detected in clinically important *Enterococcus faecium* and *E. faecalis* strains, as well as in strains of *Lactococcus garviae*, an opportunistic emerging zoonotic and human pathogen, which is also associated with different food matrices [61,62]. Among *L. pseudomesenteroides* strains, particular interest emerged from strain 4882, which displayed a mobile element 12 kb downstream of *lsa*A belonging to the IS30 family. Insertion elements of this family are quite common in LAB, as they were detected in strains of *Lactobacillus delbrueckii*, *Lactiplantibacillus plantarum*, *Leuconostoc lactis*, *Pediococcus pentosaceus* and *Oenococcus oeni* [63,64,65]. 

Presence of mobile elements flanking a putative AR gene in the genomes of these food-grade and technological *Leuconostoc* strains highlights the need to phenotypically assess the antibiotic resistance patterns, as well as the effective transferability of this trait in filter and in food mating trials [20]. In this framework, it has already been demonstrated the ability of *Leuconostoc* strains (belonging to *L. mesenteroides*) to transfer ARG conferring erythromycin resistance to recipient *E. faecalis* strains both in lab conditions and in cheese, from which these strains were isolated [20].

To understand the role of *lsa*A at the phenotypic level, the MIC values of CLI and QUD were determined by broth microdilution plates for the type strains of *L. fallax* and *L. pseudomesenteroides*. Both strains showed resistance to CLI, while *L. pseudomesenteroides* displayed resistance only towards QUD. Even though their MIC values (4 and 16 μg/mL for CLI and 8 and 2 μg/mL for QUD, respectively) are lower than those reported for *E. faecalis* strains (32–48 μg/mL for CLI and 32 μg/mL for QUD) [29], it is interesting to note that the MIC distributions for *E. faecalis* reported from the EUCAST website (https://mic.eucast.org/, checked on 2 February 2021) showed that resistant strains displayed MIC values between 4 and 8 μg/mL more frequently for QUD and between 8 and 16 μg/mL for CLI; thus, they are comparable with those observed in the two *Leuconostoc* strains. 

To date, this is the first study reporting a clindamycin-resistant *L. pseudomesenteroides* strain harboring *lsa*A; in fact, a previous work on *L. pseudomesenteroides* strains isolated from fermented table olives, clindamycin-resistant strains did not show the presence of the *lsa*A gene [21]. 

The discrepancy between the genotype and phenotype towards QUD for *L*. *pseudomesenteroides* was further investigated with the analysis of the expression of *lsa*A in LMG 11482^T^, which was found to increase in the presence of both CLI as well as QUD. As such, data obtained may suggest that ECOFF proposed for QUD for the genus *Leuconostoc*, 4 μg/mL, might be not appropriate to distinguish resistant from susceptible strains belonging to the *L. pseudomesenteroides* species. Therefore, an update of the quinupristin-dalfopristin ECOFF should be performed, analyzing the MIC values of a larger number of *L. pseudomesenteroides* isolates.

## 5. Conclusions

The results obtained in this study represent a starting point for the generation of new and more focused scientific protocols and regulatory procedures based on WGS approaches for the genotype–phenotype safety assessment of *Leuconostoc* strains employed as starter cultures, probiotic or food preservatives.

In this framework, the phenotypic data obtained (MIC values and *lsa*A expression analysis) surely open the way to a wider validation of these observations, which necessarily has to be carried out on a higher number of *L. pseudomesenteroides* and *L. fallax* strains, as well as on all the isolates (belonging to *L. lactis* and *L. falkebergense*) that were found to display the *lsa*A gene. Further phenotypic analysis should include gene cloning, knock-out vector development and MIC determination in strains with the disrupted gene as it was performed both by Singh and colleagues in 2002 to characterize *lsa*A in almost 500 strains of *Enterococcus* strains [29] and by Shi and coauthors in 2020 to characterize *lsa*D (responsible for resistance to lincosamides, streptogramins and pleuromutilins) in *Lactococcus garvieae* strains [66].

We showed that ARG searches against CARD databases are not sufficient and accurate as they could be for some pathogenic bacteria [67,68]. Therefore, the implementation of ARGs available for LAB could result in a paradigm shift from phenotype- to genotype-based assessments of the resistance for food-grade and technological bacteria in order to enrich the number of AR determinants included in the database. This research is expected to improve the effectiveness of genome sequence analysis as a tool for the prediction of AR characters even in foodborne bacteria. 

In this perspective, WGS-based approaches could be used as a tool for the surveillance of the emergence and spread of AR determinants in bacteria, providing an important initial contribution to the identification of genes potentially associated with resistance as well as relevant information about the possibility of AR genes being spread along the food chain.

## Figures and Tables

**Table 1:** Genome features of the 17 type-strains of the *Leuconostoc* genus analyzed in this study. The genome sequences of the type strains of *L. gelidum aenigmaticum* LMG 27840^T^, *L. miyukkimchii* DSM 25624^T^, *L. palmae* DSM 21144^T^, *L. rapi* DSM 27776^T^ are not publicly available.

Species	Type Strain	Isolation Source	GenBank Accession Number
*Leuconostoc carnosum*	DSM 5576^T^	Chill-stored meat	JACHGL000000000.1
*Leuconostoc citreum*	ATCC 49370^T^	Food and clinical sources	PUFH00000000.1
*Leuconostoc falkenbergense*	LMG 10779^T^	Lactic culture	BMBS00000000.1
*Leuconostoc fallax*	ATCC 700006^T^	Sauerkraut	PUFI00000000.1
*Leuconostoc gelidum* subsp. *gasicomitatum*	LMG 18811^T^	Packaged meat	FN822744.1
*Leuconostoc gelidum* subsp. *gelidum*	KCTC 3527^T^	Kimchi (a traditional Korean food made by fermentation of Chinese cabbage)	AEMI00000000.1
*Leuconostoc holzapfelii*	CCUG 54536^T^	Ethiopian coffee fermentation	JAAXPO000000000.1
*Leuconostoc inhae*	KCTC 3774^T^	Kimchi	AEMJ00000000.1
*Leuconostoc kimchii*	IMSNU 11154^T^	Kimchi	chromosome: NC_014136.1/CP001758.1 plasmid: LkipL4701: NC_014131.1/CP001753.1 LkipL4704: NC_014132.1/CP001754.1 LkipL4719: NC_014133.1/CP001755.1 LkipL4726: NC_014134.1/CP001756.1 LkipL48: NC_014135.1/CP001757.1
*Leuconostoc lactis*	DSM 20202^T^	Milk	AEOR00000000.1
*Leuconostoc litchii*	MB7^T^	Lychee	SDGY00000000.1
*Leuconostoc mesenteroides* subsp. *cremoris*	ATCC 19254^T^	Hansen’s dried starter powder	ACKV00000000.1
*Leuconostoc mesenteroides* subsp. *cremoris*	DSM 20484^T^	Missing (isolated in 1912)	chromosome: NZ_CP012009.1/CP012009.1 plasmid pDSM20484: NZ_CP012010.1/CP012010.1
*Leuconostoc mesenteroides* subsp. *Jonggajibkimchii*	DRC1506^T^	Kimchi	chromosome: NZ_CP014611.1/CP014611.1 plasmid pDRC1: NZ_CP014612.1/CP014612.1 pDRC2: NZ_CP014613.1/CP014613.1 pDRC3: NZ_CP014614.1/CP014614.1
*Leuconostoc mesenteroides* subsp. *mesenteroides*	ATCC 8293^T^	Fermenting olives	chromosome: NC_008531.1/CP000414.1 plasmid pLEUM1: NC_008496.1/CP000415.1
*Leuconostoc pseudomesenteroides*	NCDO 768^T^	Cane juice	JAAXPY000000000.1
*Leuconostoc suionicum*	DSM 20241^T^	Missing (isolated in 1972)	chromosome: NZ_CP015247.1/CP015247.1 plasmid unnamed: NZ_CP015248.1/CP015248.1

**Table 2:** Minimum inhibitory concentration (MIC) values for clindamycin (CLI) and quinupristin-dalfopristin (QUD) of 11 type strains determined through the broth microdilution method. MICs higher than the Epidemiological cut-off (ECOFF) values are reported in bold.

Species	MIC (µg/mL)	
	CLI	QUD
*Leuconostoc carnosum* CECT 4024^T^ (=DSM 5576^T^)	<0.03	1
*Leuconostoc fallax* LMG 13177^T^ (=ATCC 700006^T^)	**4**	**8**
*Leuconostoc gelidum* subsp. *gasicomitatum* CECT 5767^T^ (=LMG 18811^T^)	<0.03	1
*Leuconostoc gelidum* subsp. *gelidum* CECT 4026^T^ (=KCTC 3527^T^)	<0.03	0.5
*Leuconostoc inhae* CECT 7026^T^ (=KCTC 3774^T^)	<0.03	1
*Leuconostoc lactis* LMG 8894^T^ (=DSM 20202^T^)	<0.03	1
*Leuconostoc mesenteroides* subsp. *cremoris* LMG 6909^T^ (=ATCC 19254^T^)	0.12	1
*Leuconostoc mesenteroides* subsp. *dextranicum* LMG 6908^T^ (=ATCC 19254^T^)	0.12	0.5
*Leuconostoc mesenteroides* subsp. *mesenteroides* LMG 6893^T^ (=ATCC 8293^T^)	0.12	1
*Leuconostoc pseudomesenteroides* LMG 11482^T^ (=NCDO 768^T^)	**16**	2
*Leuconostoc suionicum* CECT 8146^T^ (=DSM 20241^T^)	0.12	1
ECOFF	1	4

## Data Availability

Publicly available datasets were analyzed in this study. Accession numbers of genomes and gene sequences used are reported in Table 1, Figure 2 and Figure 4.

## Supplementary Materials

The following are available online at https://www.mdpi.com/2076-2607/9/3/491/s1: Table S1: Putative AR genes identified in the *Leuconostoc* type strains analyzed; Figure S1: Distribution of drug efflux pumps proteins in the *Leuconostoc* type strains dataset; File S1: Analysis of the flanking regions of strains belonging to *Leuconostoc falkebergense*, *L. fallax*, “*L. garlicum*”, *L. lactis, L. pseudomesenteroides* and *Enterococcus faecalis*.
