# Supplementary material for: Exploring Antibiotic Resistance Diversity in Leuconostoc spp. by a Genome-Based Approach: Focus on the lsaA Gene
Source: Microorganisms. 2021 Feb 26;9(3):491. doi: 10.3390/microorganisms9030491 (PMC7996808; doi:10.3390/microorganisms9030491)
Supplement: Supplementary file 1 [file microorganisms-09-00491-s001.zip › Salvetti_et_al_SupplementaryMaterial/TableS1.pdf]

Species	Am	Bl	Di	Gl	Li	Li-Ma-Ox-Ph-St-Te	Li-Ma-St	Ph	Ri	St
Leuconostoc carnosum **DSM 5576^{T}**		mecI(1)	dfrA6(2), dfrA12(1), dfrA13(1), dfrA14(1), dfrA18(1), dfrA20(1), dfrA21(1), dfrA22(1), dfrA24(1), dfrA25(1), dfrA26(1), dfrA29(1), dfrA3(1), dfrA30(1), dfrA31(1), dfrA5(1), dfrC(1), dfrD(1), dfrE(1), dfrF(1), dfrG(1), dfrI(1), dfrK(1)	vanRA(6), vanRF(6), vanRM(6), vanRB(5), vanRC(5), vanRD(5), vanRE(5), vanRG(5), vanRI(5), vanRL(5), vanRN(5), vanRO(4), vanHA(3), vanHB(3), vanHF(3), vanHO(3), vanHD(2), vanHM(2), vanA(1), vanB(1), vanD(1), vanF(1), vanI(1), vanM(1), vanN(1), vanO(1), vanXYC(1), vanXYL(1), vanXYN(1)	lin(4)	vgaE(8), tvaA(3), vgaC(3), vgaD(3), vmlR(3), lmrC(2), lsaB(2), msrF(2), poxtA(2), carA(1), lsaA(1), lsaC(1), msrA(1), msrC(1), msrH(1), oleB(1), optrA(1), salA(1)	ErmD(1)	catB7(2), cat(1), catB10(1), catB11(1), catB2(1), catB3(1), catB8(1), catB9(1)	rpoB(2), rpoB2(2)	vatA(1), vatB(1), vatC(1), vatD(1), vatE(1), vatF(1), vatH(1), VatI(1)
Leuconostoc citreum **ATCC 49370^{T}**			dfrA6(2), dfrA18(1), dfrA20(1), dfrA24(1), dfrA25(1), dfrA29(1), dfrA3(1), dfrA31(1), dfrC(1), dfrD(1), dfrE(1), dfrG(1), dfrI(1), dfrK(1)	vanRF(6), vanRM(6), vanRA(5), vanRB(5), vanRC(5), vanRD(5), vanRE(5), vanRG(5), vanRI(5), vanRL(5), vanRN(5), vanHF(4), vanRO(4), vanHA(3), vanHB(3), vanHD(3), vanHM(3), vanHO(3), vanA(1), vanB(1), vanC(1), vanD(1), vanF(1), vanI(1), vanL(1), vanM(1), vanN(1), vanO(1), vanYG1(1)	lin(4)	vgaE(8), lmrC(4), vmlR(4), poxtA(3), vgaC(3), vgaD(3), lsaA(2), lsaB(2), msrE(2), msrF(2), tlrC(2), tvaA(2), msrA(1), msrC(1), optrA(1), srmB(1), vgaA(1), vgaALC(1)			rpoB(2), rpoB2(2)	
Leuconostoc falkenbergense **LMG 10779^{T}**			dfrA6(2), dfrA12(1), dfrA13(1), dfrA15b(1), dfrA16(1), dfrA21(1), dfrA22(1), dfrA25(1), dfrA26(1), dfrA29(1), dfrA3(1), dfrA31(1), dfrA9(1), dfrC(1), dfrD(1), dfrE(1), dfrF(1), dfrG(1), dfrI(1), dfrK(1)	vanRC(7), vanRF(7), vanRL(7), vanRM(7), vanRN(7), vanRA(6), vanRB(6), vanRD(6), vanRE(6), vanRG(6), vanRI(6), vanHA(5), vanRO(5), vanHB(4), vanHF(4), vanHM(4), vanHD(3), vanHO(3), vanXYL(2), vanXYN(2), vanA(1), vanB(1), vanC(1), vanD(1), vanF(1), vanI(1), vanM(1), vanN(1), vanO(1), vanSA(1), vanSG(1), vanYM(1)	lin(3)	vgaE(10), vmlR(6), msrF(5), poxtA(4), vgaD(4), carA(3), lmrC(3), tvaA(3), vgaC(3), lsaB(2), msrA(2), msrC(2), vgaA(2), lsaA(1), mel(1), msrH(1), oleB(1), optrA(1), srmB(1), vgaALC(1), vgaB(1)			rpoB(2), rpoB2(2)	
Leuconostoc fallax **ATCC 700006^{T}**			dfrA12(1), dfrA13(1), dfrA15(1), dfrA15b(1), dfrA16(1), dfrA18(1), dfrA21(1), dfrA22(1), dfrA25(1), dfrA26(1), dfrA29(1), dfrA3(1), dfrA6(1), dfrA8(1), dfrA9(1), dfrC(1), dfrD(1), dfrE(1), dfrF(1), dfrG(1), dfrI(1)	vanRM(6), vanRA(5), vanRB(5), vanRC(5), vanRD(5), vanRE(5), vanRF(5), vanRG(5), vanRI(5), vanRL(5), vanRN(5), vanRO(5), vanHA(4), vanHB(4), vanHD(4), vanHF(4), vanHM(4), vanHO(4), vanA(1), vanB(1), vanC(1), vanD(1), vanE(1), vanF(1), vanI(1), vanM(1), vanN(1), vanO(1), vanSC(1), vanSN(1), vanTrL(1), vanWB(1), vanWG(1	lin(3)	vgaE(7), carA(4), lmrC(4), msrH(4), vgaD(4), lsaA(3), lsaC(3), msrF(3), tvaA(3), vgaC(3), lsaB(2), msrA(2), oleB(2), poxtA(2), tlrC(2), vgaALC(2), vmlR(2), msrC(1), optrA(1), srmB(1), vgaA(1)			rpoB(2), rpoB2(2)	
Leuconostoc gelidum **subsp.** ***gasicomitatum*** **LMG 18811^{T}**		mecI(1)	dfrA12(1), dfrA14(1), dfrA15(1), dfrA15b(1), dfrA22(1), dfrA24(1), dfrA25(1), dfrA26(1), dfrA29(1), dfrA3(1), dfrA30(1), dfrA31(1), dfrA5(1), dfrA6(1), dfrC(1), dfrD(1), dfrE(1), dfrF(1), dfrG(1), dfrI(1), dfrK(1)	vanHF(6), vanRA(6), vanRB(6), vanRC(6), vanRF(6), vanRG(6), vanRM(6), vanHA(5), vanHO(5), vanRD(5), vanRE(5), vanRI(5), vanRL(5), vanRN(5), vanRO(5), vanHB(4), vanHD(4), vanHM(4), vanA(1), vanB(1), vanC(1), vanD(1), vanF(1), vanKI(1), vanL(1), vanM(1), vanN(1), vanO(1), vanSC(1), vanXYN(1)	lin(2)	vgaE(11), carA(5), srmB(5), vgaC(4), vmlR(4), lmrC(3), poxtA(3), salA(3), vgaD(3), msrA(2), oleB(2), tvaA(2), lsaA(1), lsaB(1), msrE(1), msrF(1), msrH(1), optrA(1)		cat(1), catB3(1), catB8(1), catB9(1), catB7(1)	rpoB(2), rpoB2(2)	vatA(1), vatB(1), vatC(1), vatD(1), vatE(1), vatF(1), vatH(1), VatI(1)
Leuconostoc gelidum **subsp.** ***gelidum*** **KCTC 3527^{T}**		mecI(1)	dfrA1(1), dfrA12(1), dfrA14(1), dfrA15b(1), dfrA18(1), dfrA24(1), dfrA25(1), dfrA26(1), dfrA29(1), dfrA3(1), dfrA30(1), dfrA31(1), dfrA5(1), dfrA6(1), dfrA9(1), dfrC(1), dfrD(1), dfrE(1), dfrF(1), dfrG(1), dfrI(1), dfrK(1)	vanHB(6), vanHF(6), vanRA(6), vanRB(6), vanRC(6), vanRF(6), vanRG(6), vanRM(6), vanHA(5), vanHO(5), vanRD(5), vanRE(5), vanRI(5), vanRL(5), vanRN(5), vanRO(5), vanHD(4), vanHM(4), vanA(1), vanB(1), vanC(1), vanD(1), vanF(1), vanKI(1), vanL(1), vanM(1), vanN(1), vanO(1), vanWB(1), vanWG(1), vanXYN(1)	lin(2)	vgaE(11), carA(5), srmB(5), lmrC(4), vmlR(4), tvaA(3), vgaC(3), vgaD(3), msrA(2), oleB(2), poxtA(2), salA(2), lsaA(1), lsaB(1), msrE(1), msrF(1), msrH(1), optrA(1), tlrC(1), vgaA(1), vgaALC(1)		cat(1), catB10(1), catB3(1), catB8(1), catB9(1), catB7(1)	rpoB(2), rpoB2(2)	vatA(1), vatB(1), vatC(1), vatD(1), vatE(1), vatF(1), vatH(1), VatI(1)
Leuconostoc holzapfelii **CCUG 54536^{T}**			dfrA12(1), dfrA13(1), dfrA18(1), dfrA20(1), dfrA21(1), dfrA22(1), dfrA26(1), dfrA3(1), dfrA31(1), dfrA3b(1), dfrC(1), dfrD(1), dfrE(1), dfrF(1), dfrG(1), dfrI(1), dfrK(1)	vanRA(7), vanRB(7), vanRC(7), vanRF(7), vanRG(7), vanRI(7), vanRL(7), vanRM(7), vanRN(7), vanHF(6), vanRD(6), vanRE(6), vanHA(5), vanRO(5), vanHB(4), vanHM(4), vanHO(4), vanHD(3), vanA(1), vanB(1), vanF(1), vanM(1), vanWB(1), vanWG(1)	lin(3)	vgaE(11), carA(6), srmB(6), vgaD(6), msrA(5), msrF(5), oleB(5), vmlR(5), msrH(4), vgaC(4), msrC(3), poxtA(3), vgaA(3), vgaALC(3), lmrC(2), lsaB(2), msrE(2), optrA(2), tlrC(2), lsaC(1), salA(1), tvaA(1)	erm40(1), ErmD(1)			
Leuconostoc inhae **KCTC 3774^{T}**		mecI(1)	dfrA12(1), dfrA14(1), dfrA15(1), dfrA15b(1), dfrA22(1), dfrA24(1), dfrA25(1), dfrA26(1), dfrA29(1), dfrA3(1), dfrA30(1), dfrA31(1), dfrA5(1), dfrA6(1), dfrC(1), dfrD(1), dfrE(1), dfrF(1), dfrG(1), dfrI(1), dfrK(1)	vanRA(6), vanRB(6), vanRC(6), vanRF(6), vanRG(6), vanRM(6), vanHF(5), vanRD(5), vanRE(5), vanRI(5), vanRL(5), vanRN(5), vanRO(5), vanHA(4), vanHO(4), vanHB(3), vanHD(3), vanHM(3), vanA(1), vanB(1), vanC(1), vanD(1), vanF(1), vanL(1), vanM(1), vanN(1), vanO(1), vanSC(1), vanXYN(1)		carA(4), srmB(4), vgaE(4), lmrC(2), poxtA(2), lsaA(1), lsaB(1), oleB(1), salA(1), tvaA(1), vgaC(1)			rpoB(2), rpoB2(2)	
Leuconostoc kimchii **IMSNU 11154^{T}**			dfrA6(2), dfrA3(1), dfrD(1), dfrF(1), dfrC(1), dfrA25(1), dfrA5(1), dfrI(1), dfrA12(1), dfrA14(1), dfrA8(1), dfrA29(1), dfrG(1), dfrA13(1), dfrA15b(1), dfrA26(1), dfrA21(1), dfrA30(1), dfrA22(1), dfrK(1), dfrE(1)	vanHM(6), vanHF(6), vanRA(6), vanRB(6), vanRC(6), vanRF(6), vanRM(6), vanHA(5), vanHB(5), vanRE(5), vanRD(5), vanRG(5), vanRL(5), vanRN(5), vanRI(5), vanRO(4), vanHD(3), vanHO(3), vanC(1), vanA(1), vanF(1), vanE(1), vanD(1), vanL(1), vanM(1), vanO(1), vanB(1), vanWB(1), vanWG(1), vanTrL(1)	lin(4)	vgaE(14), vgaALC(5), srmB(5), vgaC(5), vmlR(5), vgaA(4), lmrC(4), vgaD(4), optrA(4), msrH(3), msrF(3), tvaA(3), oleB(2), poxtA(2), lsaC(2), msrE(2), msrA(2), salA(1), lsaB(1), tlrC(1), msrC(1), carA(1)	ErmD(1), Erm39(1)	catB7(2), cat(1), catB9(1), catB8(1), catB2(1), catB3(1), catB11(1), catB10(1)	rpoB2(2), rpoB(2)	vatA(1), vatB(1), vatC(1), vatF(1), vatE(1), vatD(1), vatH(1), VatI(1)

Leuconostoc lactis **DSM 20202** **T**		mecI(1)	dfrA6(2), dfrA12(1), dfrA13(1), dfrA18(1), dfrA20(1), dfrA21(1), dfrA22(1), dfrA26(1), dfrA3(1), dfrA31(1), dfrA3b(1), dfrA8(1), dfrC(1), dfrD(1), dfrE(1), dfrF(1), dfrG(1), dfrI(1), dfrK(1)	vanHF(3), vanRA(3), vanRB(3), vanRC(3), vanRD(3), vanRE(3), vanRF(3), vanRG(3), vanRI(3), vanRL(3), vanRM(3), vanRN(3), vanRO(3), vanHA(2), vanHD(2), vanHO(2), vanHB(1), vanHM(1)	lin(1)	vgaE(5), msrA(3), srmB(3), lsaB(2), tvaA(2), vgaC(2), vgaD(2), vmlR(2), lmrC(1), lsaC(1), msrF(1), msrH(1), oleB(1), optrA(1), poxtA(1), vgaA(1), vgaALC(1)	erm40(1), ErmB(1)	
Leuconostoc litchii **MB7** **T**		mecI(2)	dfrA12(1), dfrA13(1), dfrA14(1), dfrA15(1), dfrA15b(1), dfrA16(1), dfrA17(1), dfrA21(1), dfrA22(1), dfrA24(1), dfrA25(1), dfrA26(1), dfrA27(1), dfrA29(1), dfrA3(1), dfrA30(1), dfrA5(1), dfrA6(1), dfrC(1), dfrD(1), dfrE(1), dfrF(1), dfrG(1), dfrI(1), dfrK(1)	vanRA(6), vanRC(6), vanRD(6), vanRF(6), vanRG(6), vanRI(6), vanRL(6), vanRM(6), vanRN(6), vanRB(5), vanRE(5), vanHB(4), vanHF(4), vanRO(4), vanHA(3), vanHM(3), vanHO(3), vanHD(2), vanA(1), vanB(1), vanC(1), vanE(1), vanF(1), vanG(1), vanI(1), vanL(1), vanM(1), vanN(1), vanO(1), vanSG(1), vanTrL(1), vanUG(1), vanWB(1), vanWG(1), vanYB(1)	lin(4)	vgaE(6), vgaD(4), vmlR(4), carA(3), lmrC(3), msrA(3), poxtA(3), srmB(3), tvaA(3), msrC(2), msrF(2), msrH(2), oleB(2), lsaA(1), lsaB(1), optrA(1), tlrC(1), vgaA(1), vgaALC(1), vgaC(1)	erm46(1), ErmX(1)	rpoB(2), rpoB2(2)
Leuconostoc mesenteroides **subsp.** ***cremoris*** **ATCC 19254** **T**		mecI(1)	dfrA12(1), dfrA13(1), dfrA14(1), dfrA16(1), dfrA18(1), dfrA21(1), dfrA22(1), dfrA26(1), dfrA3(1), dfrA30(1), dfrA5(1), dfrA9(1), dfrC(1), dfrD(1), dfrE(1), dfrF(1), dfrG(1), dfrI(1), dfrK(1)	vanRA(5), vanRB(5), vanRC(5), vanRD(5), vanRE(5), vanRF(5), vanRG(5), vanRI(5), vanRL(5), vanRM(5), vanRN(5), vanRO(4), vanHA(3), vanHB(3), vanHF(3), vanHO(3), vanHD(2), vanHM(2), vanA(1), vanB(1), vanC(1), vanD(1), vanE(1), vanF(1), vanG(1), vanI(1), vanL(1), vanM(1), vanN(1), vanO(1), vanSG(1), vanUG(1)	lin(3)	vgaE(9), vmlR(5), lmrC(3), lsaA(3), oleB(3), carA(2), vgaC(2), lsaB(1), msrA(1), msrF(1), msrH(1), optrA(1), srmB(1), tlrC(1), tvaA(1), vgaA(1), vgaD(1)		rpoB(2), rpoB2(2)
Leuconostoc mesenteroides **subsp.** ***dextranicum*** **DSM 20484** **T**		mecI(2)	dfrA12(1), dfrA13(1), dfrA14(1), dfrA16(1), dfrA18(1), dfrA21(1), dfrA22(1), dfrA25(1), dfrA26(1), dfrA29(1), dfrA3(1), dfrA30(1), dfrA5(1), dfrA9(1), dfrC(1), dfrD(1), dfrE(1), dfrF(1), dfrG(1), dfrI(1), dfrK(1)	vanHA(6), vanHB(6), vanRB(6), vanRE(6), vanRF(6), vanRI(6), vanRM(6), vanHF(5), vanHM(5), vanRA(5), vanRC(5), vanRD(5), vanRG(5), vanRL(5), vanRN(5), vanHD(4), vanRO(4), vanHO(3), vanA(1), vanB(1), vanC(1), vanD(1), vanE(1), vanF(1), vanG(1), vanI(1), vanL(1), vanM(1), vanN(1), vanO(1), vanSA(1), vanSG(1), vanXYL(1), vanXYN(1)	lin(2)	vgaE(8), vmlR(4), lmrC(3), msrH(3), vgaC(3), carA(2), lsaA(2), msrC(2), oleB(2), poxtA(2), tvaA(2), vgaD(2), lsaB(1), lsaC(1), msrA(1), msrF(1), optrA(1), srmB(1), tlrC(1), vgaA(1)		rpoB(2), rpoB2(2)
Leuconostoc mesenteroides **subsp.** ***jonggajibkimchii*** **DRC1506** **T**		mecI(2)	dfrA12(1), dfrA13(1), dfrA14(1), dfrA15b(1), dfrA16(1), dfrA18(1), dfrA21(1), dfrA22(1), dfrA25(1), dfrA26(1), dfrA29(1), dfrA3(1), dfrA30(1), dfrA5(1), dfrA9(1), dfrC(1), dfrD(1), dfrE(1), dfrF(1), dfrG(1), dfrI(1), dfrK(1)	vanRF(8), vanRM(8), vanHA(7), vanHB(7), vanRA(7), vanRB(7), vanRC(7), vanRE(7), vanRI(7), vanRL(7), vanRN(7), vanHF(6), vanHM(6), vanRD(6), vanRG(6), vanHD(5), vanRO(5), vanHO(4), vanA(1), vanB(1), vanC(1), vanD(1), vanE(1), vanF(1), vanG(1), vanI(1), vanL(1), vanM(1), vanN(1), vanO(1), vanSA(1), vanSG(1), vanXYL(1), vanXYN(1)	lin(2)	vgaE(8), lmrC(4), vmlR(4), carA(3), oleB(3), vgaC(3), lsaA(2), srmB(2), tvaA(2), vgaD(2), lsaB(1), lsaC(1), msrA(1), msrC(1), msrF(1), msrH(1), optrA(1), poxtA(1), tlrC(1), vgaA(1)		rpoB(2), rpoB2(2)
Leuconostoc mesenteroides **subsp.** ***mesenteroides*** **ATCC 8293** **T**		mecI(2)	dfrA12(1), dfrA13(1), dfrA14(1), dfrA16(1), dfrA18(1), dfrA21(1), dfrA22(1), dfrA25(1), dfrA26(1), dfrA29(1), dfrA3(1), dfrA30(1), dfrA5(1), dfrA9(1), dfrC(1), dfrD(1), dfrE(1), dfrF(1), dfrG(1), dfrI(1), dfrK(1)	vanHA(7), vanHB(7), vanRF(7), vanRM(7), vanHF(6), vanHM(6), vanRA(6), vanRB(6), vanRC(6), vanRE(6), vanRI(6), vanRL(6), vanRN(6), vanHD(5), vanRD(5), vanRG(5), vanHO(4), vanRO(4), vanA(1), vanB(1), vanC(1), vanD(1), vanE(1), vanF(1), vanG(1), vanI(1), vanL(1), vanM(1), vanN(1), vanO(1), vanSA(1), vanSG(1), vanXYL(1), vanXYN(1)	lin(2)	vgaE(8), vmlR(4), lmrC(3), lsaA(3), vgaC(3), carA(2), oleB(2), srmB(2), tvaA(2), vgaD(2), lsaB(1), lsaC(1), msrA(1), msrC(1), msrF(1), msrH(1), optrA(1), poxtA(1), tlrC(1), vgaA(1)		rpoB(2), rpoB2(2)
Leuconostoc pseudomesenteroides **NCDO 768** **T**		mecI(2)	dfrA6(2), dfrA12(1), dfrA13(1), dfrA15(1), dfrA15b(1), dfrA21(1), dfrA22(1), dfrA25(1), dfrA26(1), dfrA29(1), dfrA3(1), dfrA30(1), dfrA9(1), dfrC(1), dfrD(1), dfrE(1), dfrF(1), dfrG(1), dfrI(1), dfrK(1)	vanRB(8), vanRF(8), vanRM(8), vanRA(7), vanRC(7), vanRE(7), vanRG(7), vanRI(7), vanRL(7), vanRN(7), vanRD(6), vanHA(5), vanHD(5), vanRO(5), vanHB(4), vanHF(4), vanHM(4), vanHO(3), vanXYL(2), vanXYN(2), vanA(1), vanB(1), vanC(1), vanD(1), vanF(1), vanI(1), vanM(1), vanN(1), vanO(1), vanSA(1), vanSG(1), vanXYC(1), vanYM(1)	lin(5)	vgaE(17), vgaALC(7), vgaD(7), vgaA(6), vgaC(6), vmlR(6), lmrC(5), msrF(5), srmB(5), tvaA(5), carA(4), msrH(4), optrA(4), lsaA(3), oleB(3), poxtA(3), lsaB(2), msrA(2), msrC(2), tlrC(2), lsaC(1), msrE(1), salA(1), vgaB(1)		rpoB(2), rpoB2(2)
Leuconostoc suionicum **DSM 20241** **T**	cpaA(1)	mecI(2)	dfrA12(1), dfrA13(1), dfrA14(1), dfrA15b(1), dfrA18(1), dfrA21(1), dfrA22(1), dfrA25(1), dfrA26(1), dfrA29(1), dfrA3(1), dfrA30(1), dfrA5(1), dfrA7(1), dfrA9(1), dfrC(1), dfrD(1), dfrE(1), dfrF(1), dfrG(1), dfrI(1), dfrK(1)	vanHA(8), vanHB(7), vanHM(7), vanRA(7), vanRF(7), vanRM(7), vanHF(6), vanRB(6), vanRG(6), vanRI(6), vanRL(6), vanHD(5), vanRC(5), vanRD(5), vanRE(5), vanRN(5), vanHO(4), vanRO(4), vanA(1), vanB(1), vanC(1), vanD(1), vanF(1), vanG(1), vanI(1), vanL(1), vanM(1), vanN(1), vanO(1), vanSA(1), vanSG(1), vanXYL(1), vanXYN(1)	lin(2)	vgaE(8), carA(4), lmrC(4), vmlR(4), tvaA(3), vgaC(3), lsaA(2), msrC(2), srmB(2), tlrC(2), vgaD(2), lsaB(1), lsaC(1), mel(1), msrF(1), msrH(1), oleB(1), optrA(1), poxtA(1), salA(1), vgaA(1)		rpoB(2), rpoB2(2)
