# Supplementary material for: Exploring Antibiotic Resistance Diversity in Leuconostoc spp. by a Genome-Based Approach: Focus on the lsaA Gene
Source: Microorganisms. 2021 Feb 26;9(3):491. doi: 10.3390/microorganisms9030491 (PMC7996808; doi:10.3390/microorganisms9030491)
Supplement: Supplementary file 1 [file microorganisms-09-00491-s001.zip › Salvetti_et_al_SupplementaryMaterial/Salvetti_Suppl_20210210.docx]

Research Pape

Exploring antibiotic resistance diversity in Leuconostoc spp. by a genome-based approach: focus on the gene lsaA

Elisa Salvetti^1a^, Ilenia Campedelli^2a^, Ilaria Larini^1^, Giada Conedera^1^, Sandra Torriani^1^*

**Table S1.** Putative AR genes identified in the *Leuconostoc* type strains analyzed

**Figure S1.** Distribution of drug efflux pumps proteins in the *Leuconostoc* type strains dataset.

**File S1.** Analysis of the flanking regions of strains belonging to *Leuconostoc* *falkebergense*, *L. fallax*, ‘*L. garlicum’*, *L. lactis,* *L. pseudomesenteroides,* end *Enterococcus faecalis*.

.
