# Supplementary figures and images for: Exploring Antibiotic Resistance Diversity in Leuconostoc spp. by a Genome-Based Approach: Focus on the lsaA Gene
Source: Microorganisms. 2021 Feb 26;9(3):491. doi: 10.3390/microorganisms9030491 (PMC7996808; doi:10.3390/microorganisms9030491)

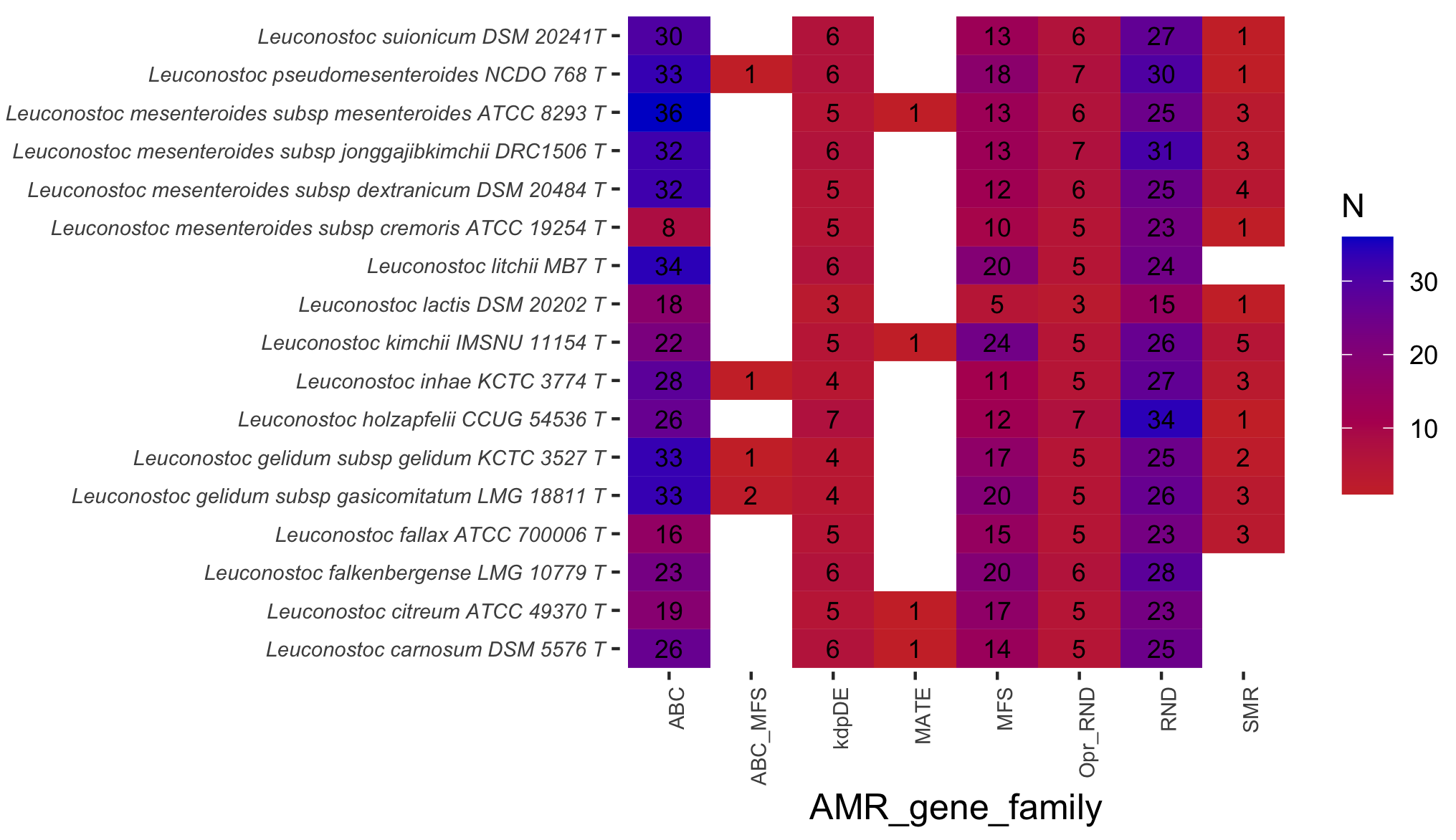

Supplement: Supplementary file 1 [file microorganisms-09-00491-s001.zip › Salvetti_et_al_SupplementaryMaterial/FigureS1.png]
